# A Case of Vernal Keratoconjunctivitis With Growth Hormone Deficiency

**DOI:** 10.7759/cureus.30615

**Published:** 2022-10-23

**Authors:** Atsuki Fukushima, Hitoshi Tabuchi

**Affiliations:** 1 Ophthalmology, Tsukazaki Hospital, Himeji, JPN; 2 Ophthalmology, Hiroshima University, Hiroshima, JPN

**Keywords:** vernal keratoconjunctivitis, stature, growth hormone deficiency, growth hormone, allergy

## Abstract

Hormonal abnormalities are considered to play a role in the development of vernal keratoconjunctivitis (VKC). However, little is known whether about growth hormone (GH) is related to VKC development. The patient was an 11-year-old male with VKC treated with 0.1% betamethasone eye drops and 0.1% cyclosporin eye drops. The papillary growth of both superior and inferior palpebral conjunctiva worsened, and masses started to appear at the lower palpebral margin. He was referred to our hospital and was treated with 0.1% tacrolimus and 0.1% fluorometholone. Six weeks later, the condition improved remarkably. At this timepoint, we noticed the patient’s short stature and asked again about his past history. Two years ago, he was diagnosed with GH deficiency (GHD), which had been treated with somatropin in a pediatric clinic. Thus, it is necessary to keep in mind the possibility of GHD when treating VKC patients.

## Introduction

Hormonal abnormalities have been reported to be frequently noted in VKC patients [1]. The role of hormones in the development of VKC remains to be elucidated. However, it was postulated that sex hormones exert a proinflammatory effect to recruit eosinophils into the conjunctiva [2]. In contrast, regarding the relationship between growth hormone (GH) and VKC, we were able to confirm only one report [3] within the scope of our investigation. Here, we report a case of VKC in a male who was also treated with GH deficiency (GHD).

## Case presentation

**Figure 1:**
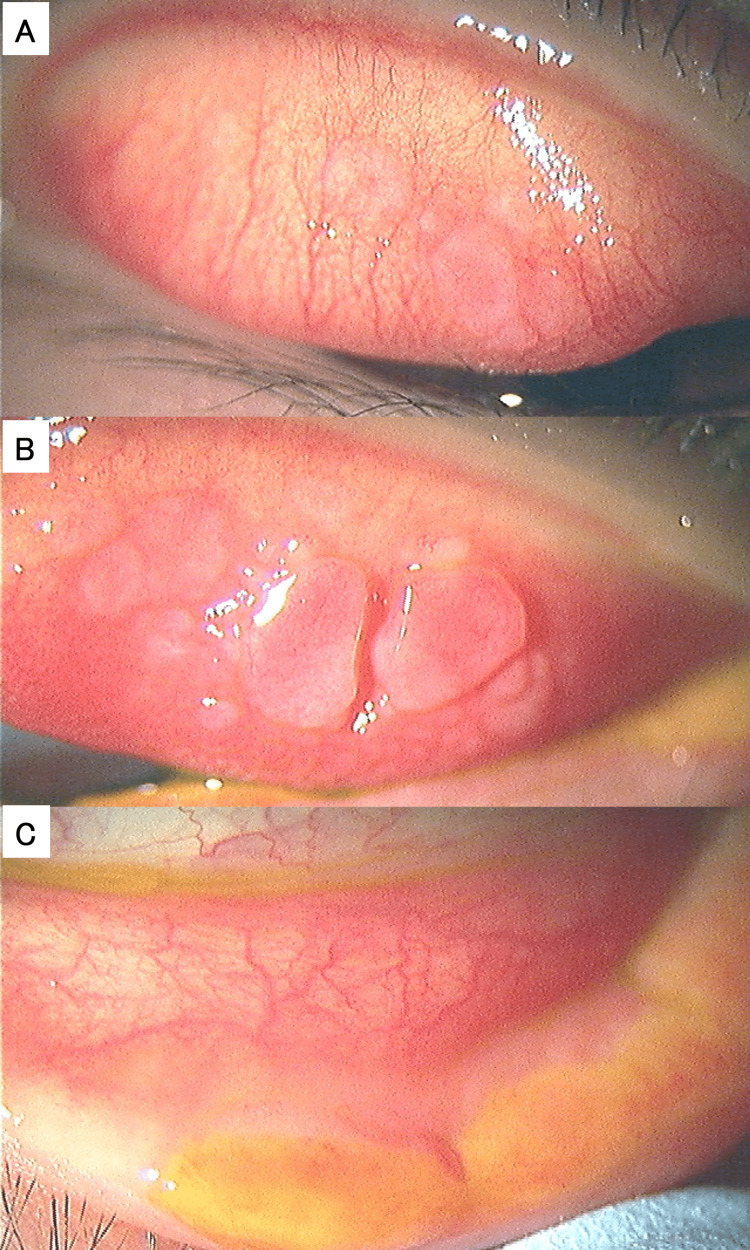
Slit lamp microscopic photographs upon initial presentation. (A) Upper palpebral conjunctiva of the right eye. (B) Upper palpebral conjunctiva of the left eye. (C) Lower eyelid margin of the left eye.

An 11-year-old male developed atopic dermatitis at the age of 2 and was treated for this in the pediatrics department of another institution. Blood sampling tests detected IgE for Japanese cedar pollen, Japanese cypress, mites, house dust, grasses, and animal epithelium. He was diagnosed with vernal keratoconjunctivitis (VKC) several years ago, which was treated with 0.1% betamethasone eye drops and 0.1% cyclosporin eye drops in another ophthalmology clinic. From July 2021, the papillary growth of both superior and inferior palpebral conjunctiva worsened. Furthermore, masses started to appear at the palpebral margin, which gradually worsened, prompting referral to our hospital on February 2022. Giant papillae were found in the conjunctiva of the upper eyelid, and tumors were found in the margin of the lower left eyelid (Figure 1).

**Figure 2:**
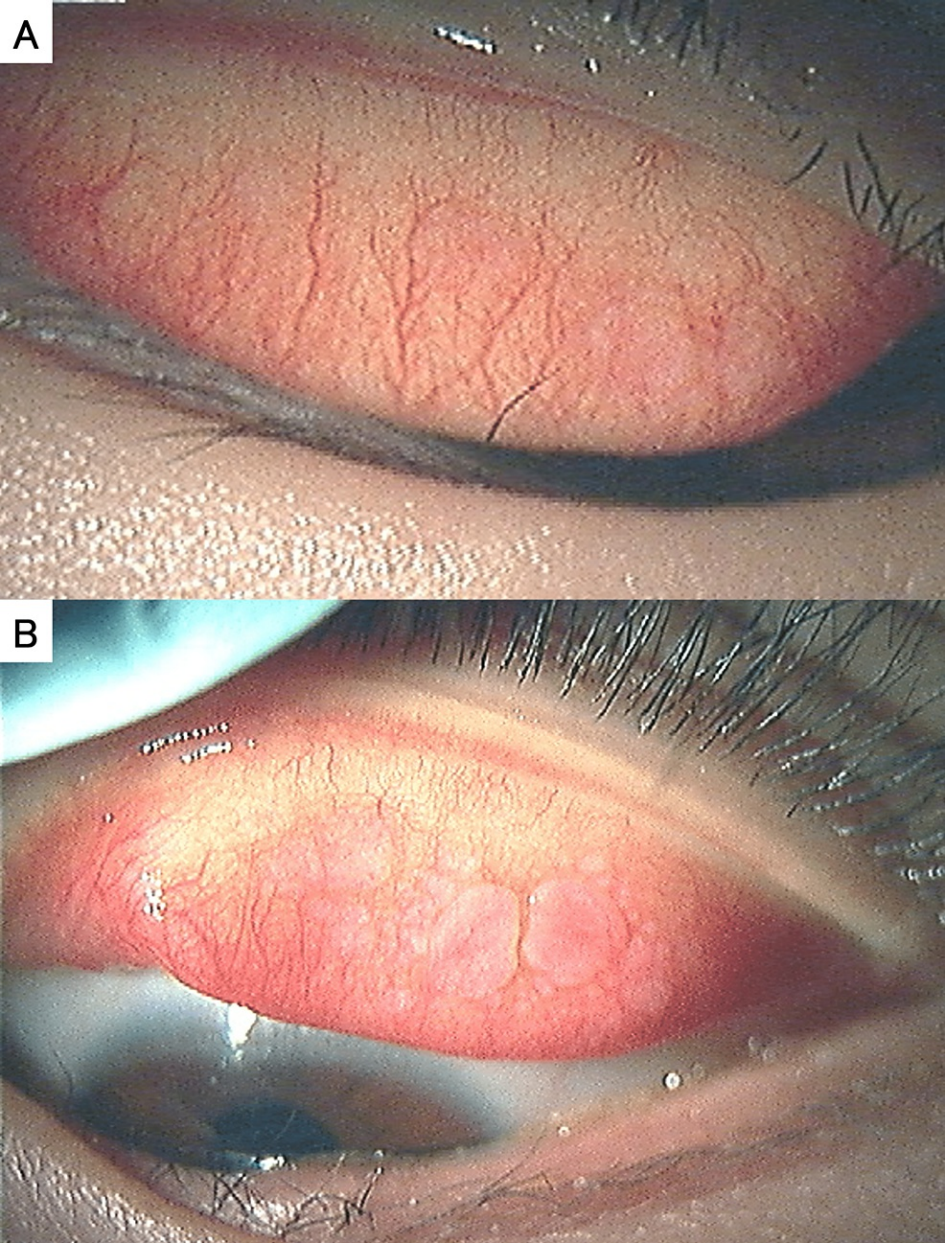
Slit lamp microscopic photographs six weeks after treatment. (A) Upper palpebral conjunctiva of the right eye. (B) Upper palpebral conjunctiva and lower lid margin of the left eye.

Conjunctival scraping cytology confirmed eosinophils, and the treatment for VKC was judged to be inadequate. Then, the eye drops were changed to 0.1% tacrolimus instillation twice a day and 0.1% fluorometholone instillation four times a day. Six weeks after the change in treatment regimen, the condition improved remarkably, as shown in Figure 2.

At this timepoint, we noticed the patient’s short stature and asked again about his past history. In the department of pediatrics, he was diagnosed with GHD, which was treated with somatropin (12 mg, subcutaneous injection), starting on May 23, 2020. From May 2020 to March 2022, the patient’s height was -2.0 standard deviations (SD), whereas his weight was -1.5 SD.

## Discussion

Six cases of VKC with GHD have been reported [3]. All cases showed significant growth retardation regarding both height and weight when diagnosed with GHD (-2.05 to -2.78). Three cases had follow-ups until adulthood, and the effect of GH treatment was confirmed on the stature growth. However, there was no detailed description of the course and treatment of VKC [3], probably because the authors investigated from the viewpoint of the endocrine field, not from the viewpoint of the ophthalmology. Thus, it is unclear how GHD and its treatment affected the disease course of VKC [3].

Hormonal abnormalities have been reported to be frequently noted in VKC patients [1]. It was postulated that sex hormones exert a proinflammatory effect to recruit eosinophils into the conjunctiva, thus leading to the development of VKC [2]. Regarding the involvement of GH on allergic inflammation, it was reported that GHD was diagnosed in four (16%) patients out of 25 in nonallergic group and in 13 (30.9%) patients out of 42 in nickel-allergic group [4]. Thus, GHD may play a role, at least in part, in the development of allergic inflammation, such as VKC.

It was reported that baseline C-reactive protein (CRP) levels were higher than healthy controls and GH therapy reduced CRP levels [5]. More recently, another group reported that GH therapy reduced IL-6 and CRP levels, although not significantly [6]. The authors discussed that the reasons for insignificant reduction of IL-6 and CRP were perhaps because subjects were prepubertal and follow-up was relatively short (six months). Therefore, it is currently unknown how giving GH to people with GHD affects inflammation, especially VKC. The anti-inflammatory effects of GH for GHD will be clarified by studying data from patients in which it was provided.

## Conclusions

Our report supports the claim made in the previous article that when treating VKC patients, it is important to bear the likelihood of GHD in mind. To identify the connection between VKC and GHD, more examples of VKC with GHD must be gathered.
